# All-Trans Retinoic Acid in Combination with Primaquine Clears *Pneumocystis* Infection

**DOI:** 10.1371/journal.pone.0053479

**Published:** 2013-01-04

**Authors:** Guang-Sheng Lei, Chen Zhang, Shoujin Shao, Hsin-Wei Jung, Pamela J. Durant, Chao-Hung Lee

**Affiliations:** 1 Department of Pathology and Laboratory Medicine, Indiana University School of Medicine, Indianapolis, Indiana, United States of America; University of Alabama at Birmingham School of Medicine, United States of America

## Abstract

*Pneumocystis* pneumonia (PcP) develops in immunocompromised patients. Alveolar macrophages play a key role in the recognition, phagocytosis, and degradation of *Pneumocystis*, but their number is decreased in PcP. Our study of various inflammatory components during PcP found that myeloid-derived suppressor cells (MDSCs) accumulate in the lungs of mice and rats with *Pneumocystis* pneumonia (PcP). We hypothesized that treatment with all-trans retinoic acid (ATRA), a metabolite of vitamin A, may effectively control *Pneumocystis* (Pc) infection by inducing MDSCs to differentiate to AMs. In rodent models of PcP, we found that 5 weeks of ATRA treatment reduced the number of MDSCs in the lungs and increased the number of AMs which cleared Pc infection. We also found that ATRA in combination with primaquine was as effective as the combination of trimethoprim and sulfamethaxazole for treatment of PcP and completely eliminated MDSCs and Pc organisms in the lungs in two weeks. No relapse of PcP was seen after three weeks of the ATRA-primaquine combination treatment. Prolonged survival of Pc-infected animals was also achieved by this regimen. This is the very first successful development of a therapeutic regimen for PcP that combines an immune modulator with an antibiotic, enabling the hosts to effectively defend the infection. Results of our study may serve as a model for development of novel therapies for other infections with MDSC accumulation.

## Introduction

*Pneumocystis* pneumonia (PcP) is a common opportunistic disease in immunocompromised hosts, such as patients with AIDS [1], [2] and those with other predisposing immune deficiencies including acute lymphoblastic leukemia, severe combined immunodeficiency syndrome, Hodgkin’s disease, rhabdomyosarcoma, Wegener’s granulomatosis, collagen vascular disease, and primary metastatic tumor in the central nervous system [3]. The use of immunosuppressive drugs in organ transplantation patients [4] and anti-TNF-α monoclonal antibodies, such as infliximab [5] and adalimumab [6], in patients with rheumatoid arthritis may also result in PcP.

Drugs that are commonly used to treat PcP include the combination of trimethoprim and sulfamethoxazole (TMP-SMX, also referred to as Septra, Bactrim, and Co-trimoxazole), pentamidine, dapsone, atovaquone, and the combination of clindamycin and primaquine. Among these, TMP-SMX and pentamidine are considered first line drugs [7]. TMP-SMX is the most effective drug for treatment and prevention of PcP. Unfortunately, many patients fail therapy with TMP-SMX due to toxicity or resistance. In a study of 38 AIDS patients with PcP, only five patients completed TMP-SMX therapy. Twenty-nine (76%) of these patients had drug toxicity, and 19 (50%) had to be switched to other therapies. The adverse effects of TMP-SMX found in this study included rash (86%), fever (77%), neutropenia (66%), thrombocytopenia (26%), and transaminase elevation (31%) [8]. In a study of 962 European AIDS patients with PcP treated with TMP-SMX, 22% of the patients were switched to other regimens [7].

The mechanisms of PcP pathogenesis are largely unknown. Lung damage during PcP is mainly due to inflammatory responses mediated by CD8 T-cells [9]–[11]. CD4 T-cells have also been shown to play such role in Pc-related immune reconstitution inflammatory syndrome [12]. A characteristic feature of PcP is that alveolar macrophages (AMs) are defective in phagocytosis [13]–[15]. The expression of the antizyme inhibitor is greatly increased in AMs [16]. Since antizyme inhibitor stabilizes ornithine decarboxylase, the key enzyme in polyamine synthesis, and promotes the import of exogenous polyamines, polyamine levels in AMs are elevated leading to increased rate of apoptosis and decreased number of AMs [16], [17]. Pc infection also causes reduced production of calmodulin, resulting in a defect in the dimerization of iNOS and thus decreased production of nitric oxide by AMs [18]. The expression of the PU.1 gene in AMs is also decreased [19]. Since PU.1 regulates the expression of many macrophage receptors [19]–[23], this finding partially explains the defect in phagocytosis of AMs during PcP. Very recently, we found that myeloid-derived suppressor cells (MDSCs) accumulate in the lung during PcP [24].

MDSCs are a heterogeneous population of bone marrow-derived myeloid progenitor cells and immature myeloid cells and are immunosuppressive [25]. All-trans retinoic acid (ATRA), one form of vitamin A-derived retinoids, has been shown to stimulate MDSCs to differentiate to dendritic cells and macrophages [26], [27], and administration of therapeutic concentrations of ATRA can substantially decrease the number of MDSCs in tumor-bearing mice and in patients with cancer, improving their antigen-specific response of T-cells [28], [29]. We hypothesize that ATRA treatment can push the MDSCs in the lungs to differentiate to functional AMs which may clear Pc infection. In this study, we tested the effects of ATRA alone and in combination with an 8-aminoquinoline primaquine for treatment of PcP.

## Materials and Methods

### Rodent Models of PcP

C57BL/6 mice and Sprague Dawley rats were obtained from Harlan (Indianapolis, IN). All animals used in this study were female, with body weights of 18–20 g in mice and 120–140 g in rats. Animal studies were approved by the Indiana University Animal Care and Use Committee and carried out under the supervision of veterinarians. Immunosuppression of mice was achieved by intraperitoneal injection of 0.3 mg anti-CD4 mAb (clone GK1.5, Harlan, Indianapolis, IN) once a week until the mice were sacrificed. Three days after the initial injection, mice were transtracheally instilled with 2×10^6^ of Pc organisms in 50 µl sterile PBS. Rats were immunosuppressed with 1.8 mg/ml dexamethasone in drinking water. One week after initiation of immunosuppression, rats were transtracheally instilled with 2×10^6^ of Pc organisms in 200 µl sterile PBS. The Pc organisms used as inoculum were obtained from heavily infected lungs and isolated as previously described [30]. Tetracycline (0.74 g/L) was added to the drinking water to prevent bacterial infections. Immunosuppressed-uninfected animals were used as controls. For treatment, TMP-SMX (TMP, 50 mg/kg/day and SMX, 250 mg/kg/day), ATRA (5 mg/kg/day in 8% DMSO), and primaquine (PMQ) (2 mg/kg/day in water) were given orally once a day starting from 3 weeks post Pc inoculation for mice and 2 weeks post Pc inoculation for rats. ATRA at 5 mg/kg/day is equivalent to 15 mg/m^2^/day [31]. TMP-SMX (Septra) was purchased from Hi-Tech Pharmacal (Amityville). Both ATRA and PMQ were obtained from Sigma-Aldridge (St. Louis, MO).

### Histopathology Examination and Evaluation of Infection

Animals were anesthetized by intramuscular injection of ketamine cocktail (ketamine hydrochloride, 80 mg/ml; acepromazine, 1.76 mg/ml; atropine, 0.38 µg/ml) and then sacrificed by cardiac exsanguination. The left lung was removed from each animal, inflated with buffered 10% formalin, fixed overnight, and then embedded in paraffin. Five-micrometer histologic sections were stained with H&E for evaluation of lung inflammation and architecture. Histologic lung sections stained with Grocott’s methenamine silver (GMS) and modified Wright-Giemsa stained lung impression smears were examined under light microscope to determine organism burden.

### Isolation of Total BAL Cells

Lungs were lavaged with sterile saline (5 ml for rats and 1 ml for mice at a time) through an intratracheal catheter until a total of 50 ml of lavage fluid from each rat or 10 ml from each mouse was recovered as described previously [32]. The bronchoalveolar lavage fluid (BALF) was centrifuged at 300×*g* for 10 min to pellet BAL cells. The pelleted cells were washed twice with PBS and then resuspended in PBS at 1×10^6^ cells per ml.

### Morphological Analysis of BAL Cells

One hundred microliters of a cell suspension (3×10^4^ cells/ml) was loaded into a cytospin chamber and spun for 5 min at 500 rpm (Cytospin 2, Shandon). Slides were air-dried at room temperature for 5 min and stained with Giemsa using the LeukoStat staining kit (Fisher Scientific).

### Flow Cytometry Analysis

BAL cells were obtained from uninfected or Pc-infected animals. After incubating in 5% bovine serum albumin for 1 hr, the cells were stained with specific fluorescence-labeled antibodies including anti-mouse CD11b, Gr-1, and CD11c; and anti-rat CD11bc and His48 (BioLegend) on ice for 1 hr. Separate sets of cells were stained with phycoerythrin (PE) or FITC-labeled IgG isotype control antibody. After washing twice with 4 ml PBS, the stained cells were examined with a BD FACSCalibur flow cytometer (BD Biosciences), and the flow cytometry data thus generated were analyzed with the FlowJo software (Tree Star, Ashland, OR).

### Statistic Analysis

Comparisons were made between the mean values of the treatment and control groups or between two treatment groups by the unpaired Student’s t test with a two-tail distribution. Comparisons between three or more treatment groups were made by one ANOVA. Survival rates between groups were compared by Mantel-Cox test and Gehan-Breslow-Wilcoxon test. A p value<0.05 was considered significant.

## Results

### ATRA Eliminated MDSC and Cleared Pc Infection in Mice after 5 Weeks of Treatment

**Figure 1:**
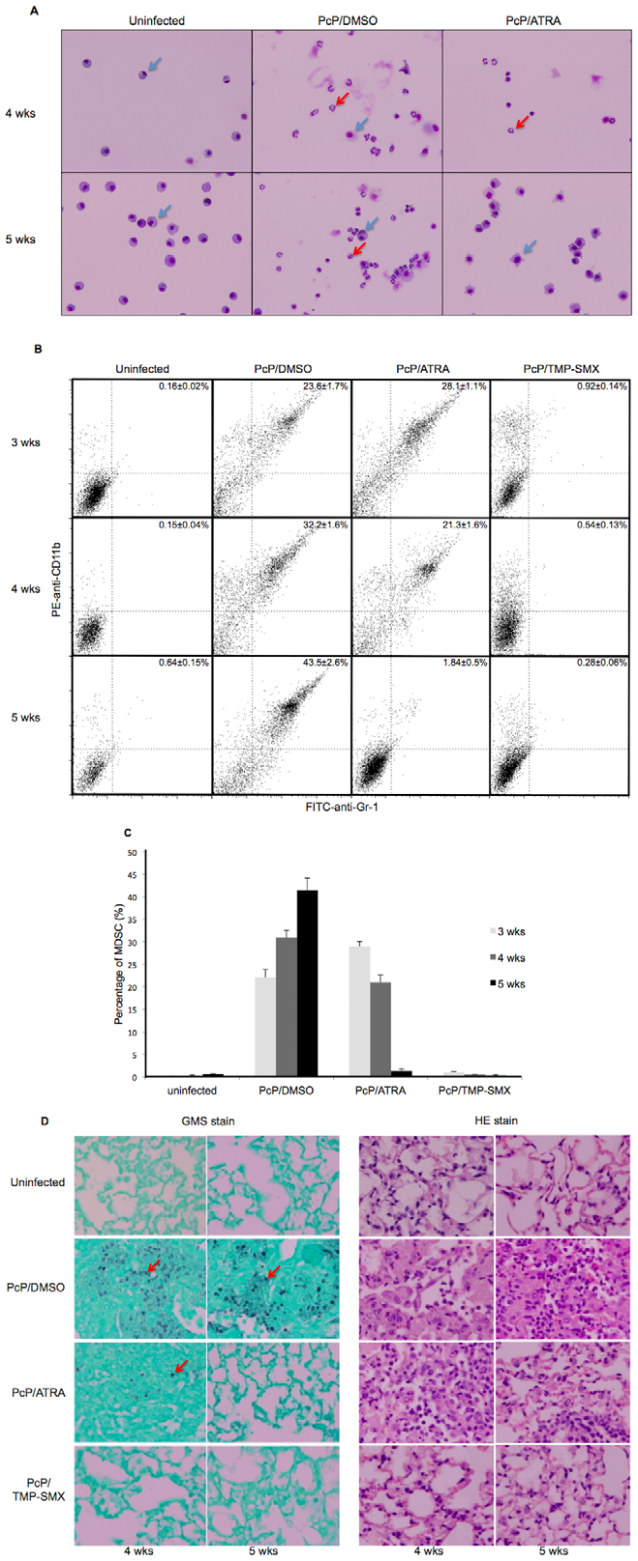
Effects of ATRA treatment on MDSCs and Pc infection in mice. Immunosuppressed, Pc-infected mice were treated with vehicle control 8% DMSO, ATRA, or TMP-SMX for 3, 4, and 5 weeks as indicated in the figure. Treatment was initiated 3 weeks after Pc inoculation. (**A**) Giemsa-stained BAL cells cytospun on slides. Red arrows indicate MDSC-like cells, and blue arrows denote alveolar macrophages. (**B**) Representative flow cytograms of BAL cells from mice in each group at each time point. The percentage of Gr-1^+^/CD11b^+^ cells in each sample is shown in the right upper quadrant of each flow cytogram. (**C**) Percentage of Gr-1^+^/CD11b^+^ cells in BALF, as determined by flow cytometry. Data are means ± S. D. of 3–6 mice in each group at each time point. (D) Histologic sections of the lungs stained with GMS (left) and H&E (right). Red arrows indicate GMS-stained Pc organisms. Images are representative of 3–6 mice in each group at each time point. Microscope magnification: 40X.

To investigate the potential therapeutic effect of ATRA on PcP, immunosuppressed C57BL/6 mice were transtracheally inoculated with Pc and then treated with ATRA at 5 mg/kg/day starting 3 weeks post Pc inoculation. This group of mice was referred to as the PcP/ATRA group (Fig. 1). A separate group of Pc-infected mice, referred to as the PcP/DMSO group, were treated with 8% DMSO, which was the vehicle used to dissolve ATRA, in the same manner to serve as controls. Three mice each in uninfected, PcP/DMSO, and PcP/ATRA groups were sacrificed and lavaged every week for 5 weeks after initiation of ATRA treatment. The number of MDSCs in the BALF of each mouse was determined by flow cytometry and by morphological examinations of BAL cells cytospun on slides. The severity of Pc infection was determined by examining lung sections stained with H&E for histology and GMS for Pc organism load.

As shown in Fig. 1A, cells with ring-like nuclei characteristic of MDSCs were seen in the BALF from untreated PcP mice (PcP/DMSO group). These cells were not present in the BALF from uninfected mice and were greatly reduced in numbers in the BALF from Pc-infected mice after 4 weeks of ATRA treatment. Few such cells were observed in the BALF from uninfected mice or Pc-infected mice treated with ATRA for 5 weeks.

Flow cytometry studies of BAL cells revealed the presence of a population of Gr-1^+^/CD11b^+^ MDSCs in the BAL samples from Pc-infected mice but not in those from uninfected mice. The percentage of these cells in PcP mice with 3 weeks or less ATRA treatment (28.1%) was similar to that (23.6%) in PcP mice treated with DMSO only (Fig. 1B and Fig. 1C). While the percentage of MDSCs increased by approximately 20% 5 weeks post DMSO treatment, it decreased by 26% after Pc-infected mice had been treated with ATRA for 5 weeks.

Histology examinations showed that the alveoli of Pc-infected mice were filled with exudates, Pc organisms, and inflammatory cells at 4 and 5 weeks post DMSO treatment (Fig. 1D). The lung inflammation and architecture of PcP mice were improved after 4 weeks of ATRA treatment and became close to normal after 5 weeks of ATRA treatment (Fig. 1D). The organism load as revealed by GMS staining was greatly reduced after 4 weeks of ATRA treatment. No GMS stained Pc organisms were seen in the lung sections from PcP mice that had been treated with ATRA for 5 weeks (Fig. 1D).

### Combination of ATRA with PMQ Eliminated MDSCs and Cleared Pc Infection in 2 Weeks

**Figure 2:**
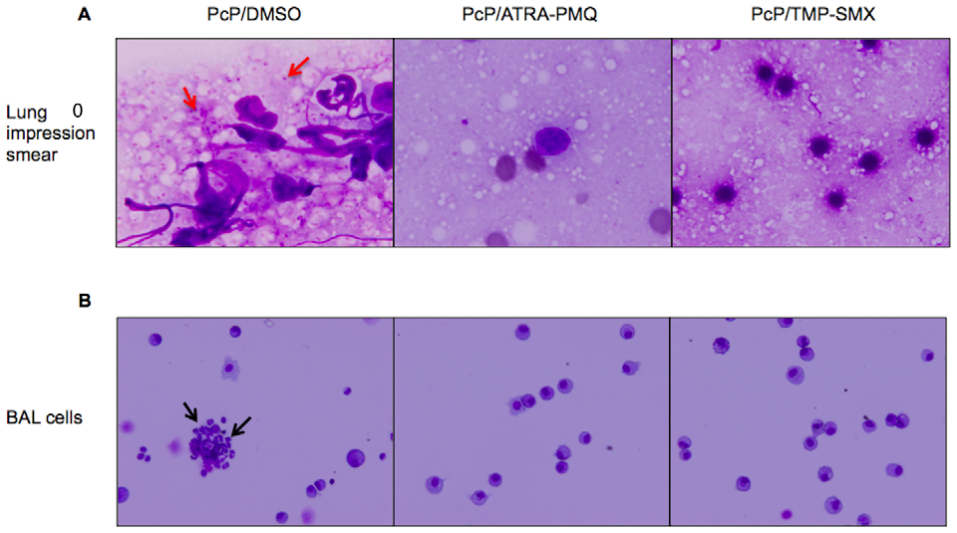
Effects of ATRA-PMQ combination on MDSCs and Pc infection in mice. Immunosuppressed, Pc-infected mice were given vehicle control 8% DMSO, ATRA-PMQ, or TMP-SMX for 2 weeks. Treatment was initiated 3 weeks after Pc inoculation. (A) Lung impression smear stained with Giemsa. Red arrows indicate nuclei of Pc organisms. Magnification: 100X. (B) Giemsa-stained BAL cells cytospun on slides. Black arrows indicate MDSC-like cells. Microscope magnification: 40X. Images are representative of 3–6 mice of each group.

The results described above showed that ATRA treatment could cure PcP; however, it required at least 5 weeks of treatment. Since ATRA itself is not known to have any antimicrobial activity, we tested whether ATRA in combination with PMQ would control Pc infection more efficiently than ATRA alone. Immunosuppressed mice with Pc infection for 3 weeks were treated with ATRA (5 mg/kg/day) plus PMQ (2 mg/kg/day) or TMP-SMX (TMP, 50 mg/kg/day and SMX, 250 mg/kg/day) for 2 weeks. BAL cells cytospun on slides and lung impression smears were examined morphologically for the presence of MDSCs and Pc organisms, respectively. Surprisingly, the combination of ATRA and PMQ combination worked as effectively as TMP-SMX; it cleared the infection and also eliminated MDSCs in the lungs in two weeks in all PcP mice (Fig. 2).

**Figure 3:**
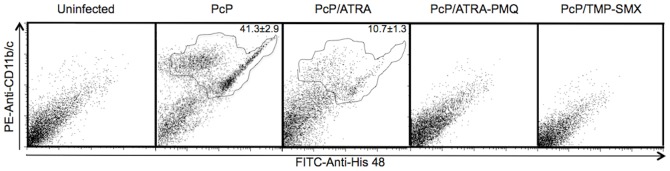
Effects of ATRA, ATRA-PMQ, and TMP-SMX on MDSCs in rats. Immunosuppressed, Pc-infected rats were given vehicle control 8% DMSO, ATRA, ATRA-PMQ, or TMP-SMX for 4 weeks. Treatment was initiated 2 weeks after Pc inoculation. BAL cells were examined by flow cytometry. The percentage of His48^+^/CD11bc^+^ MDSCs in each sample is shown in the right upper quadrant of each flow cytogram.

To determine whether the combination of ATRA and PMQ also works in other animals, the same regimen was tested in rats. Immunosuppressed Sprague Dawley rats with Pc infection for 2 weeks were treated with ATRA, ATRA-PMQ, or TMP-SMX for 4 weeks. A group of Pc-infected rats treated with 8% DMSO was used as untreated control. Since MDSCs in rats are His48^+^/CD11bc^+^, the numbers of MDSCs in the BALF were determined by flow cytometry using anti-His48 and anti-CD11bc antibodies. The severity of Pc infection was determined by examining lung sections stained with H&E for histology and GMS for Pc organism load. As shown in Fig. 3, a significant number of His48^+^/CD11bc^+^ MDSCs were present in the BAL fluid from untreated PcP rats. MDSCs were greatly reduced in number (10.7%) after 4 weeks of ATRA treatment, and were completely disappeared after 4 weeks treatment with ATRA-PMQ or TMP-SMX. Histological examination of lung sections revealed that lung inflammation and architecture of Pc-infected rats were greatly improved after 4 weeks of ATRA treatment and were close to normal after 4 weeks treatment with ATRA-PMQ or TMP-SMX, similar to those observed in mice (data not shown).

### ATRA and ATRA-PMQ Treatments Increased the Number of AMs

**Figure 4:**
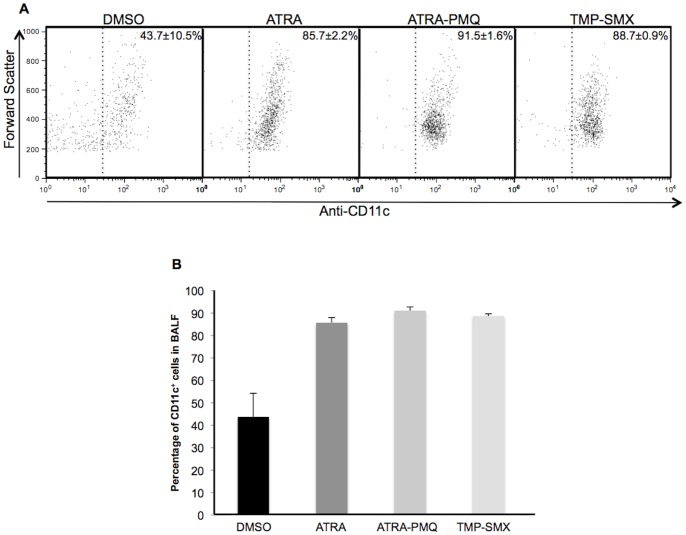
Increased number of CD11c^+^ BAL cells after ATRA, ATRA-PMQ, or TMP-SMX treatment. Immunosuppressed, Pc-infected mice were given vehicle control 8% DMSO, ATRA, ATRA-PMQ, or TMP-SMX for 5 weeks. Treatment was initiated 3 weeks after Pc inoculation. BAL cells were collected and analyzed by flow cytometry with fluorescence labeled anti-mouse CD11c antibody. (**A**) Representative flow cytograms of BAL cells from animals of each group. (**B**) Percentage of CD11c^+^ BAL cells in BALF, as determined by flow cytometry. Data are means ± S. D. of 3–6 mice in each group.

As described above, the number of AMs is decreased during PcP. To investigate whether these drug treatments would increase the number of AMs, *Pneumocystis*-infected mice (3–6 mice per group) were treated with DMSO, ATRA, ATRA-PMQ, or TMP-SMX for 5 weeks as described above. BAL cells of untreated and treated PcP mice were collected and analyzed by flow cytometry with fluorescence-labeled anti-mouse CD11c antibody, as CD11c has been shown to be a marker of AMs [12], [33]. As seen in Fig. 4A and 4B, the number of CD11c^+^ cells was greatly increased after treatment of PcP mice with ATRA, ATRA-PMQ, or TMP-SMX. This result suggests that ATRA or ATRA-PMQ treatment converts MDSCs in the lungs of PcP mice to AMs.

### ATRA-PMQ Treatment Completely Eradicated Pc Organisms in the Lung

**Figure 5:**
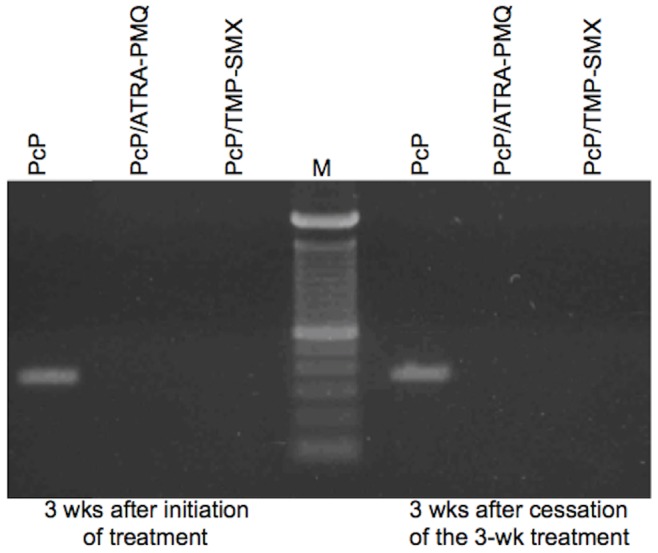
No relapse of PcP after cessation of ATRA-PMQ treatment. Immunosuppressed, Pc-infected mice were treated with ATRA-PMQ or TMP-SMX for 2 weeks and then examined for the presence of Pc at 3 weeks after initiation of treatment and at 3 weeks after cessation of the three-week treatment. Lung tissues were subjected to Pc mitochondrial rRNA PCR, and the PCR products were electrophoresed on a 1% agarose gel.

To determine whether ATRA-PMQ treatment completely eradicates Pc organisms from the lung, PcP mice were treated with the combination for 2 weeks and then examined for signs of relapse 3 weeks after cessation of the treatment. PcP mice treated with TMP-SMX were used as controls. Examination of BAL cells from these animals showed that MDSCs that were seen in infected mice were not present in the BALF from PcP mice after ATRA-PMQ or TMP-SMX treatment. Similarly, Pc organisms were seen in lung impression smears of untreated PcP mice, but not in those of ATRA-PMQ or TMP-SMX treated mice. Since microscopic examination of Pc organisms may not have sufficient sensitivity, lung tissues of untreated or ATRA-PMQ or TMP-SMX treated PcP mice were subjected to mitochondrial rRNA PCR [34], [35], and a 340-bp PCR product was detected from the lungs of untreated PcP mice but not from those of treated at the end of the 3-week treatment or at 3 weeks after cessation of the treatment (Fig. 5).

### Prolonged Survival in Animals with PcP Treated with ATRA-PMQ

**Figure 6:**
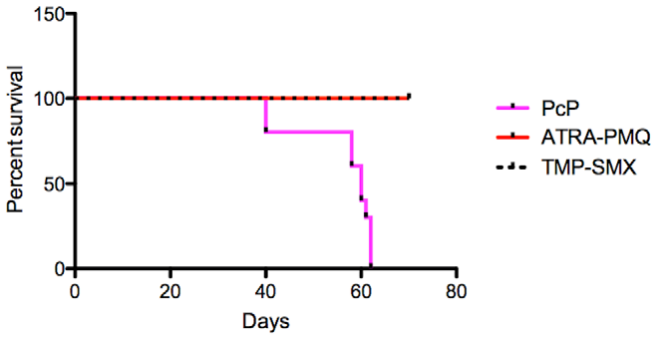
Survival curves of PcP rats treated with DMSO, ATRA-PMQ, or TMP-SMX. Animals were sacrificed when agonal or when the study was concluded. The percentage of animals survived on each day is plotted. Results are from 10 animals of each condition.

To investigate whether the ATRA-PMQ combination therapy prolongs the survival of animals with PcP, Pc-infected rats (10 in each treatment group) were treated with ATRA-PMQ or TMP-SMX daily, starting from 2 weeks post Pc inoculation and continuing through the entire period of the study. A separate group of Pc-infected rats treated with DMSO only was used as untreated control. The experiment was terminated when all animals in the control group were moribund and sacrificed. As shown in Fig. 6, PcP rats treated with ATRA-PMQ or TMP-SMX had significantly prolonged survival, compared with those of untreated group (p<0.05). At days 40, 58, 60, and 61, the numbers of untreated PcP rats that survived were 8, 6, 4, and 3, respectively. At day 62, all the remaining untreated PcP rats had to be sacrificed, whereas all 10 rats in each of the ATRA-PMQ or TMP-SMX treatment group survived for the entire 70-day period of the study.

## Discussion

Most PcP drug developments either target Pc metabolic pathways or cell wall synthesis. Because Pc cannot be continuously cultured, our understanding of Pc proliferation and metabolism is inadequate. Therefore, very little success in the development of new therapies for PcP has been made. The combination of clindamycin and PMQ as a regimen for PcP was developed in 1988 [36]; no new successful treatments have been developed since then, and current PcP treatment and prophylaxis rely on drugs developed decades ago.

In this study, we employed a novel approach to treat PcP by converting immuno-suppressive cells to immuno-protective ones so that the hosts became able to effectively defend the infection. This approach is based on our recent finding that Pc infections result in the accumulation of MDSCs in the lungs [24]. The observation that these cells suppressed the proliferation of T-cells [24] strongly suggest that they are not neutrophils as neutrophils do not have this function [37]. We found that treatment of PcP mice with ATRA for five weeks cured the disease as evidenced by the disappearance of Pc organisms (Fig. 1D) and MDSCs (Fig. 1A–C) in the lungs and greatly reduced lung inflammation (Fig. 1D). This finding further suggest that the MDSCs we observed are not neutrophils. If they were neutrophils, ATRA treatment would not eliminate them as they are terminally differentiated. Since this treatment was effective in both mice and rats with PcP (Fig. 1 and 3), it is very likely that it will also work for humans although the existence of MDSCs in the lungs of patients with PcP remains to be demonstrated.

Although ATRA treatment of Pc-infected mice or rats resulted in the clearance of the infection, it took five weeks. In contrast, the conventional TMP-SMX treatment controlled Pc infection in less than 3 weeks. In order for ATRA to become useful for treatment of PcP, a better efficacy is needed. We hypothesized that ATRA in combination with a certain antibiotic may be more effective and therefore tested PMQ for its potential synergistic effect with ATRA. Surprisingly, the combination of ATRA and PMQ was as effective as TMP-SMX for therapy of PcP and cleared the infection in two weeks, as compared to five weeks of treatment with ATRA alone.

Since PMQ by itself has no significant effect on PcP [36], the major therapeutic activity of this regimen is likely due to ATRA which presumably pushes MDSCs to further differentiate to alveolar macrophages that clear the infection. This postulation is supported by the result showing that the number of CD11c^+^ cells in the BALF from ATRA or ATRA-PMQ treatment of PcP mice was significantly increased (Fig. 4) and that of MDSCs was greatly decreased (Fig. 2). MDSCs are a heterogeneous population of bone marrow-derived myeloid progenitor cells and immature myeloid cells. In health, these cells quickly differentiate into granulocytes, macrophages, or dendritic cells. MDSC differentiation is blocked in certain pathological conditions, such as cancer, various infectious diseases, sepsis, trauma, bone marrow transplantation, and some autoimmune diseases [25]. ATRA has been shown to unblock this differentiation allowing MDSCs to be converted to macrophages and dendritic cells [26]. The role of PMQ in this combination therapy may be to prevent the proliferation of Pc, thus enabling alveolar macrophages to eradicate them as PMQ can interfere with the microbial electron transport system via the generation of quinone metabolites and superoxides [38]. Since alveolar macrophages are known to be defective in phagocytosis during PcP [13]–[15], it is possible that the newly formed alveolar macrophages due to ATRA treatment are fully functional or that ATRA treatment also activates the phagocytic activity of preexisting alveolar macrophages. These possibilities remain to be investigated.

Although the combination of ATRA and PMQ can be an effective regimen for therapy of PCP, it is by no means perfect as both ATRA and PMQ have adverse effects. In a phase I trial of ATRA on patients with various cancers including lung, head and neck, mesothelioma, colon, breast, melanoma, approximately 25% patients developed cheilitis, skin reactions, headache, nausea, vomiting, or transient elevation of transaminase and triglyceride levels [39]. ATRA is being used to treat acute promyelocytic leukemia (APL). The major adverse effect of ATRA in APL patients is retinoic acid syndrome, which is characterized by fever, weight gain, elevated white blood cells, respiratory distress, interstitial pulmonary infiltrates, pleural and pericardial effusion, dyspnea, episodic hypotension, or acute renal failure. However, these adverse effects can be effectively suppressed by steroids [40]. The recommended ATRA dose for APL is 45 mg/m^2^/day which is much greater than the dose (15 mg/m^2^/day) we used to treat PcP in mice and rats. In this study, only one dose each of ATRA (5 mg/kg/day) and PMQ (2 mg/kg/day) was used. Various doses of ATRA and PMQ are being tested to determine their optimal therapeutic concentrations, especially when they are used in combination.

Although ATRA has significant adverse effects at the doses used as an anticancer drug, the proof of the concept that ATRA can be used to treat PcP will provide a rationale to test other compounds such as liposomal ATRA which is less likely to cause retinoic acid syndrome [40]. Another approach is to use compounds such as vitamin D which also has the ability to promote the differentiation of MDSCs with much less toxicity than ATRA [41]. There have also been attempts to develop ATRA analogues that are less toxic with better or the same efficacy as ATRA. One such example is the development of Am80 which has been shown to be effective in APL patients relapsed from ATRA-induced remission and in the induction of neuronal differentiation [42]. The main adverse effect of PMQ is hemolytic anemia in patients deficient of glucose-6 phosphate dehydrogenase or glutathione synthase [38]. Another adverse effect of PMQ is methemoglobinemia due to auto-oxidation of the hemoglobin iron core. Similar to ATRA, the proof of the concept will provide a rationale to test other PMQ analogues such as aablaquine and tafenoquine that are in the final stages of clinical trials against *Plasmodium vivax* and *P. falciparum* [38]. An advantage of using PMQ for therapy is that it rarely induces resistance [38].

Using ATRA to convert MDSCs to alveolar macrophages is one form of immune modulation. Although PcP is a disease of immune dysfunction, treatment of PcP by immune modulation has not been widely investigated. Bhagwat et al. [43] showed that PcP mice treated with anti-CD3 antibody exhibit a rapid and dramatic reduction in inflammatory lung injury. Wang et al. treated PcP mice with the anti-inflammatory drug sulfasalazine and found that it reduced pulmonary inflammation and enhanced CD4+ T cell-dependent alveolar macrophage phagocytosis [12]. These approaches differ from ours in that they target lung inflammation, whereas our approach converts MDSCs to alveolar macrophages to clear the organisms, thus eliminating the original cause of lung inflammation.

In addition to providing an alternative treatment for PcP, our study can serve as a model for development of new therapies for other diseases. MDSC accumulation has been reported in many other chronic infectious disease conditions, such as toxoplasmosis [44], leishmaniasis [45], candidiasis [46], and helminthiasis [47], [48]. Eliminating MDSCs in these diseases may similarly improve host defense and help control the infections. It is conceivable that improving host defense mechanisms alone may not be completely effective. However, the combination of both immune enhancement and antimicrobial approaches may prove to be ideal. In addition, the enhancement of host defense mechanisms may also reduce the dose of antimicrobials required and thus minimize their adverse effects. It is also possible that in combination with ATRA, other drugs such as dapsone and atovaquone that are less active but less toxic than TMP-SMX will become ideal for therapy of PcP. This will be an enormous advancement in PCP therapy, as different patients may require a different therapy due to genetic variations or adverse reactions. The use of ATRA and PMQ combination as an alternative treatment for PCP will eliminate the potential risk of hypersensitivity to the SMX component of TMP-SMX. This will be a significant contribution to the treatment of PCP as approximately 10% people of a general population are allergic to sulfa drugs [49].

In summary, we have developed a novel therapy for PcP using animal models. This method challenges all the current PcP therapy paradigms. This is also the first ever regimen that may be used as an alternative to TMP-SMX which is currently the most effective drug for PcP. This new approach presumably enables the hosts to defend against the infection by making MDSCs differentiate to alveolar macrophages. It is very likely that further development of this regimen will make it useful for treatment of PcP in humans. Proving the feasibility of this approach will transform antimicrobial therapy and encourage more studies on MDSCs and development of therapies targeting MDSCs that are also a major component of the pathogenesis of illnesses such as cancer, autoimmune diseases, and other microbial diseases.
